# Legume-Derived Bioactive Peptides in Type 2 Diabetes: Opportunities and Challenges

**DOI:** 10.3390/nu15051096

**Published:** 2023-02-22

**Authors:** Kanghong Hu, Huizhong Huang, Hanluo Li, Yanhong Wei, Chenguang Yao

**Affiliations:** Sino-German Biomedical Center, Hubei Provincial Key Laboratory of Industrial Microbiology, Cooperative Innovation Center of Industrial Fermentation (Ministry of Education & Hubei Province), Key Laboratory of Fermentation Engineering (Ministry of Education), National “111” Center for Cellular Regulation and Molecular Pharmaceutics, Hubei University of Technology, Wuhan 430068, China

**Keywords:** bioactive peptide, legume, type 2 diabetes (T2D), hypoglycemic peptide

## Abstract

Diabetes mellitus is a complex disorder characterized by insufficient insulin production or insulin resistance, which results in a lifelong dependence on glucose-lowering drugs for almost all patients. During the fight with diabetes, researchers are always thinking about what characteristics the ideal hypoglycemic drugs should have. From the point of view of the drugs, they should maintain effective control of blood sugar, have a very low risk of hypoglycemia, not increase or decrease body weight, improve β-cell function, and delay disease progression. Recently, the advent of oral peptide drugs, such as semaglutide, brings exciting hope to patients with chronic diabetes. Legumes, as an excellent source of protein, peptides, and phytochemicals, have played significant roles in human health throughout human history. Some legume-derived peptides with encouraging anti-diabetic potential have been gradually reported over the last two decades. Their hypoglycemic mechanisms have also been clarified at some classic diabetes treatment targets, such as the insulin receptor signaling pathway or other related pathways involved in the progress of diabetes, and key enzymes including α-amylase, α-glucosidase, and dipeptidyl peptidase-IV (DPP-4). This review summarizes the anti-diabetic activities and mechanisms of peptides from legumes and discusses the prospects of these peptide-based drugs in type 2 diabetes (T2D) management.

## 1. Introduction

Diabetes is a metabolic disease characterized by a persistent over-normal level of blood glucose that causes impressive morbidity and mortality worldwide [1]. Persistent hyperglycemia imposes damage on other organs, such as the eye, heart, kidney, and skin, as well as the nervous system, and is strongly correlated with a myriad of diabetes-related complications [2]. Diabetes typically develops when the pancreas does not create enough insulin, or the organs involved in glucose metabolic regulation do not effectively respond to the insulin [3]. Type 1 diabetes (T1D, 5–7%), type 2 diabetes (T2D, 90%), and gestational diabetes (GD, 2–3%) are the three most common forms of diabetes [4]. T1D is a destructive autoimmune disease of pancreatic β-cells, leading to a deficiency of insulin. T2D is the most prevalent kind of diabetes in the general population, and is frequently caused by inadequate insulin production or insulin resistance [5,6]. GD is another type of diabetes that is caused by abnormal glucose tolerance and diabetes during pregnancy [7,8]. The global prevalence of diabetes shows that 537 million adults were troubled by diabetes in 2021, and the diabetic population is estimated to rise to 643 million (11.3%) in 2030 and to 783 million (12.2%) in 2045 [9]. In recent years, the incidence of T2D cases has accelerated rapidly, and it is predicted that the T2D cases may reach 700 million in 2045 [9].

Given the grim reality of T2D, several strategies including appropriate exercise, reasonable diet, and pharmacotherapy have been applied to control postprandial hyperglycemia to prevent the development of the disease. However, pharmacotherapy is the most effective method, and is favored by T2D patients. Up to now, existing drugs such as insulin and its analogues, metformin, sulfonylurea, glucagon-like peptide-1 (GLP-1) receptor agonist, dipeptidyl peptidase IV (DPP-4) inhibitor, sodium-glucose cotransporter-2 (SGLT-2) inhibitor, α-glucosidase inhibitor, etc., have been approved for the treatment of T2D. However, because no drug has yet been identified to cure T2D, nearly all T2D patients have a lifelong dependence on glucose-lowering medications. It is worth noting that recent studies have highlighted that pretreatment via the intake of special food can significantly reduce the prevalence of T2D for high-risk groups, resulting in the minimalization of the economic impact of T2D treatment [8,10].

Biopeptides are easily absorbed food-derived protein segments that play crucial roles in controlling or pretreating metabolism-related chronic diseases, such as the mild T2D subtype [11]. Recently, legume-derived peptides, such as native peptides and hydrolytic peptides from legume proteins, have been reported to prevent or treat T2D in animal models and in clinical studies. Mechanically, these peptides regulate glucose metabolism via several ways, e.g., increasing insulin secretion, improving the sensitivity of the body’s response to insulin, and inhibiting the activities of key enzymes (α-amylase, α-glucosidase, DPP-4) [12]. Aglycin family members, which are highly conserved peptides containing 37 amino acids extracted from soybeans, can resist gastric and intestinal protease degradation and exhibit insulin-like activities [13]. Legume protein hydrolysates (also called oligopeptides), always containing 2–20 amino acids produced by enzymatic hydrolysis, microbial fermentation, or food processing, have potential anti-diabetic activity because they interact with the catalytic site of the key enzymes [12,14,15]. Researchers are increasingly concerned with the development of legume-derived hypoglycemic peptide drugs and oral-specific medical foods for the control and treatment of T2D. This review summarizes the anti-diabetic activity and hypoglycemic mechanism of legume-derived peptides, which provides an option for the development of oral hypoglycemic drugs.

## 2. T2D and Its Therapy

### 2.1. The Aberrant Glucose Metabolism in T2D

The normal regulation of glucose metabolism is determined by a feedback loop consisting of pancreatic β-cells and insulin-responding tissues (such as the liver, skeletal muscle, and adipocytes) [5]. Pancreatic β-cells, located in the islets of Langerhans, lower postprandial blood glucose through glucose-dependent insulin secretion [16]. Insulin stimulates the liver, skeletal muscles, and adipocytes to transform glucose, amino acids, and fatty acids for energy storage. When insulin resistance occurs, the insulin-responding tissues and organs cannot sensitively utilize glucose. Hence, more insulin is needed to maintain the standard glucose tolerance, which may cause the overwork and dysfunction of islet β-cells [5]. β-cells’ dysfunction results in a relative lack of insulin secretion, which causes the development of hyperglycemia or T2D [6,17].

T2D is characterized by a relative lack of insulin secretion or insulin resistance occurring in the insulin-responding organs. The current multiple drugs of T2D work mainly by reversing several physiological indexes to lower blood glucose, including an increase of insulin secretion (pancreas islet β-cells), a decrease of glucagon secretion (pancreas islet α-cells), an improvement of neurotransmitter dysfunction (brain), a decrease of hepatic glucose production (liver), an increase of peripheral glucose absorption and deceleration of lipolysis (muscle and adipose tissue), a decrease of renal reabsorption of glucose (kidney), an increase of incretin effect and decrease of glucose absorption (intestinal), an improvement of balance of gut microbiota (colon) [18]. An early start and a combination of multiple therapy drugs can bring benefits to T2D patients.

### 2.2. Clinical Therapeutic Drugs and Their Targets in T2D

Some anti-diabetic drugs targeting specific tissues and organs have been approved. The definite targets of these drugs are mainly α-amylase, α-glucosidase, dipeptidyl peptidase-4 (DPP-4), glucagon-like peptide-1 (GLP-1), gastric inhibitory peptide (GIP), gastrointestinal target hormone peptide (PYY), glucose transporters (GLUTs), and sodium-dependent glucose cotransporters (SGLTs) (Table 1) [5,19,20,21]. Insulin and its analogs are the most common injectable diabetes drugs for patients with severe pancreatic dysfunction. Oral anti-diabetic drugs mainly include metformin, sulfonylureas, glinides, thiazolidinediones (TZDs), DPP-4 inhibitors, SGLT-2 inhibitors, α-amylase inhibitors, and α-glucosidase inhibitors. The current medications can lessen the harm to the body brought on by hyperglycemia by regulating blood sugar levels and increasing insulin sensitivity [22].

Insulin, the most important regulator of metabolism, reduces blood sugar by preventing hepatic glucose synthesis and promoting intracellular glucose uptake by fat and muscle cells. Insulin and its analogues have a short action period and a risk of minimally invasive infection and other side effects, such as hypoglycemia, resistance, allergy, edema, and lipodystrophy [23]. The market for peptide-based medicines has been firmly established by the creation of human insulin utilizing recombinant technology, as well as other synthetic hormones [4]. Oral insulin is superior to other forms of insulin delivery because it follows the normal physiological pathway, that is, through the portal vein circulation of the liver, first reaching the liver and later the peripheral tissues [24].

Metformin, an oral medication, is used to reduce blood sugar levels in people with non-insulin-dependent diabetic mellitus. It promotes the uptake and utilization of glucose in adipocytes and muscles and improves insulin sensitivity by reducing glucose synthesis in the liver [12]. Metformin has good security, but 5–15% of the administrated patients are still troubled by side effects, such as diarrhea, nausea, indigestion, and other gastrointestinal symptoms [20]. TZDs also ameliorate insulin resistance in adipocytes, liver, and muscle [25]. Rosiglitazone and pioglitazone, peroxisome proliferator-activated receptor c (PPARc) agonists of TZDs, act as insulin sensitizers that bring benefits by maintaining long-term glycemic balance [25]. Presently, TZDs are rarely considered for the treatment of T2D due to side effects, such as cardiac issues, edema, and weight gain.

Sulfonylureas are currently used as second-line drugs for T2D or in combination with other medications. They promote insulin secretion by blocking K+ channels in pancreatic tissue, limiting gluconeogenesis, reducing the insulin clearance rate in the liver, and reducing fatty acid decomposition [26,27]. First-generation and second-generation sulfonylureas are the two categories. The tolbutamide and chlorpropamide sulfonylureas are first-generation sulfonylureas. Glyburide (also known as glibenclamide), glipizide, glimepiride, and gliclazide are second-generation sulfonylureas. These days, second-generation sulfonate drugs are used in clinical settings more frequently. All sulfonyl medications have a common side effect called hypoglycemia, along with less serious side effects like headache, nausea, dizziness, allergic reactions, and weight gain [6]. Meglitinides, non-sulfonylureas insulin secretagogues, also target pancreatic β-cell tissue, stimulating the pancreatic β-cells to secrete insulin, without hypoglycemic risk [26].

SGLT-2 inhibitors are oral hypoglycemic medications that lower blood sugar by blocking renal glucose reabsorption. They prevent about 90% of the filtered glucose reabsorption by directly inactivating the SGLT-2, a glucose transporter located in the proximal tubules’ first section [20,28]. SGLT-2 inhibitors include dapagliflozin, canagliflozin, empagliflozin, ertugliflozin, and sotagliflozin. They also have side effects, such as urinary and genital infection risk (6.4%) and ketoacidosis risk [6]. Glucose reabsorption is blocked in the kidney when SGLT-2 is inhibited, resulting in insulin-independent glucose lowering as well as a reduction in weight and blood pressure [20].

GLP-1 receptor agonists, GLP-1 analogues, and incretin mimics are common hypoglycemic drugs. Injectable GLP-1 receptor agonists include exenatide, liraglutide, lixisenatide, albiglutide, and dulaglutide. They control the blood glucose balance via the enhancement of insulin release and suppression of excessive glucagon secretion by activating the GLP-1 receptor [7]. Exedin-4 is one of the most promising GLP-1 receptor agonists currently utilized to treat diabetes, and is injected twice daily [29]. Liraglutide is a once-daily GLP-1 analogue, whose homology of amino acids sequence with human GLP-1 is 97% [30]. The modification of a fatty acid at position 26, and the replacement of lysine with arginine at position 34, result in an extension of the plasma half-life of liraglutide (13 h), an increase of self-association, and a resistance to digestion of DPP-4 [31,32]. In 2019, semaglutide (Rybelsus^®^), the first oral hypoglycemic peptide drug, was approved by the U.S. Food and Drug Administration (FDA) to improve glycemic control in adults with T2D [33]. The combination of semaglutide and a pharmaceutical sodium 8-[(2-hydroxybenzoyl)amino]octanoate (SNAC), an absorption enhancer, leads to a better solubility of semaglutide and an increased resistance to degradation at a low pH value [31]. Despite being combined with SNAC, the oral bioavailability of Rybelsus^®^ is only 0.4–1% [30].

DPP-4 inhibitors are oral medications that extend the half-lives of endogenous incretins by preventing the DPP-4 enzyme from digesting incretins [28,34]. These medications include sitagliptin, vildagliptin, saxagliptin, linagliptin, and alogliptin. The hypoglycemic effect of DPP-4 inhibitors is characterized by glucose concentration dependence. Therefore, they generally may not cause hypoglycemia.

Mounjaro (tirzepatide) is a “first-in-class” injectable drug that activates both GIP and GLP-1 receptors, controlling hyperglycemia through a dual mechanism of action [35,36]. Imeglimin, another “first-in-class” oral tablet, can improve insulin secretion disorders with a novel mechanism of action targeting mitochondrial bioenergetics [37]. It rebalances respiratory chain activity, resulting in less reactive oxygen species formation and the prevention of mitochondrial permeability transition pore opening [37]. Chiglitazar, a PPAR complete agonist, is a new oral drug approved by China’s national medical products administration for T2D treatment in 2021; it can activate three subunits of PPAR (α, γ, and δ) [38,39].

α-glucosidase inhibitors (acarbose, miglitol, voglibose) and α-amylase inhibitors are the first-line drugs for T2D. They lower blood glucose levels by slowing down the digestion rate and inhibiting intestinal glucose absorption [40]. Their main side effect is gastrointestinal reaction.

Over the past few decades, there has been a significant increase in therapy alternatives, but none of them has prevented the decline of β-cell function. The current drugs available to treat T2D listed in Table 1 have side effects, and most of them are administered by injection. As a result, many scientists have made efforts to create alternative delivery methods, such as transdermal, oral, nasal, and ocular delivery. Due to the higher safety and compliance, the oral route remains the most appealing option for T2D patients. Currently, various oral peptide drugs have reached clinical trials, including oral insulin, calcitonin, parathyroid hormone, and vasopressin. The approval of an oral formulation of the GLP-1 receptor agonist semaglutide for the treatment of T2D represents a significant landmark in the development of oral peptide drugs [33].

## 3. Legume-Derived Peptides and Their Anti-Diabetic Activity

The legume is the fruit or seed of plants of the legume family, which are used for food, including lentils (*Lens culinaris*), common beans (*Phaseolus vulgaris*), broad beans (*Vicia faba*), dry peas (*Pisum sativum*), chickpeas (*Cicer arietinum*), cowpeas (*Vigna sinensis*), and mung beans (*Vigna radiata*) [41]. Legumes are an excellent source of good quality protein (20–45% protein), providing high amounts of the essential amino acids such as lysine and leucine, and lower amounts of methionine and tryptophan, complex carbohydrates, dietary fiber, unsaturated fats, vitamins, and essential minerals for the human diet [42,43].

The intake of legumes brings many benefits and positive effects for physical health, especially on metabolic syndromes, such as cardiovascular protection and the regulation of glucose and lipid metabolism [11,44]. Legume proteins can be converted into a variety of peptides that control vital biological activities, especially those involved in the endocrinology of living organisms [45,46]. Several observations of clinical statistics indicate that intake of legumes (lentils, chickpeas, and soybeans) or their protein significantly lowers the risk of T2D among Caucasians and Xanthoderms in different countries [47,48,49,50]. The relationship between the risk of T2D and the intake of legumes is listed in Table 2.

Given that legumes and their proteins benefit T2D patients, various legume-derived peptides are identified as anti-hyperglycemia agents due to their metabolic regulating bioactivity in vivo or in vitro. Plant-derived peptides have been shown to have hypoglycemic effects as well, which overcome security hurdles and yield an efficient treatment for T2D [4,51]. These agents can be divided into native proteins, peptides, and protein hydrolysates (hydrolytic peptides) [4,52]. The native proteins and peptides can be directly extracted and purified from legume storage or germinating seeds. Hydrolytic peptides, also called oligopeptides, are a mixture derived from the legume protein by digestion of various enzymes, fermentation of microorganisms, or mechanical cutting. Several studies have demonstrated the potential anti-diabetic activity of native preparations and hydrolysates in vitro, in vivo, or in clinical trials [53,54,55].

Animal models are the most common approaches to investigate the anti-diabetic activity of drugs in vivo, including streptozotocin (STZ) and high-fat diet (HFD)-induced diabetic mice [14,56,57], KKA^y^ diabetic mice [58], T1D Sprague Dawley (SD) rats [14,59], STZ-induced Wistar rats [60,61], HFD-C57BL/6J mice [15], and alloxan-induced diabetic mice [62]. The potential anti-diabetic benefits of legume hydrolysates and fractions or synthesized pure peptides have been investigated by several cell line models. Glucose consumption activities were evaluated by human HepG2 [15,57,59,63,64], Caco-2 [64], 3T3-L1 [64,65], and INS-1E cells [14,66]. Furthermore, Rat L6 skeletal muscle cells [67], Min6 cell NCI-H716 [65], and human embryo kidney 293 cell models [65] were used to evaluate the activation of the insulin signaling pathway. The legume-derived peptides related to anti-diabetic activities, experimental models, and anti-diabetic mechanisms are summarized in Table 3.

### 3.1. Native Legume Peptides

The primary elements of legume seeds are proteins and peptides generated by the connection of amino acids via peptide bonds. Native legume peptides (aglycin, vglycin and soymorphin-5) have been reported as anti-T2D agents due to their bioactivities in decreasing blood sugar levels, improving insulin sensitivity and glucose tolerance, and promoting the proliferation of β-cells (Table 3).

Aglycin, a biopeptide containing 37 amino acid residues, was identified as matching to the residues 27–63 of plant albumin 1 B precursor (PA1B) from pea seeds [56,77]. Aglycin has a high affinity with a 43 kDa basic 7S globulin protein [78]. This protein binding mode is similar to the interaction of insulin and insulin receptor; therefore, aglycin is also called leginsulin [79]. Structurally, it is made up of six cysteines embedded in three disulfide bonds (C3-C20, C7-C22, and C15-C32), which are highly conserved and can resist the degradation of pepsin, trypsin, and Glu-C protease [56,57]. Aglycin was initially thought to be a leginsulin that participates in plant signal transduction to control growth and differentiation [80]. Subsequently, Chen’s group found that the oral administration of aglycin in diabetic mice effectively controlled hyperglycemia by improving the insulin-signaling pathway and enhancing glucose uptake in peripheral tissues [77]. Furthermore, a long-term administration of aglycin benefited the impaired pancreatic restoration and insulin secretion [56].

Vglycin, another leginsulin, is highly similar to aglycin in amino acid sequence and structural domain [57]. It may function with a similar mechanism to aglycin, which could be useful in T2D treatment for restoring impaired insulin signaling, glucose tolerance, and pancreatic function [14]. Other leginsulin variants identified in different cultivars, as well as their bacteria-expressed recombinants, also exert insulin-like activities in myotube-like differentiated L6 and C2C12 cells [13]. Thus, leginsulins, such as aglycin and vglycin, could be considered as potential orally administrated drugs to be developed.

Soymorphin-5 (YPFVV), a µ-opioid agonist with five amino acids, is derived from soybean β-conglycinin β-subunit, and has anxiolytic-like activity [58]. It exhibited good glucose lowing activity in diabetic KKA^y^ mice [58]. Soymorphin-5 lowered blood glucose levels in the glucose tolerance test (GTT) and insulin tolerance test (ITT) after acute intraperitoneal administration, implying that soymorphin-5 might increase insulin sensitivity in KKA^y^ mice [58]. Soymorphin-5 benefits diabetic improvement by increasing plasma insulin levels and decreasing glucose and triglyceride (TG) levels. However, des-Tyr-soymorphin-5 (PFVV) loses the µ-opioid and anxiolytic-like activities, as well as their metabolic regulating activity [58].

### 3.2. Legume Proteins

Legume seed proteins are the main dietary source of plant protein, in which anti-nutritional substances include lectins, hydrolase inhibitors, ribosome-inactivating proteins (RIPs), and allergens [81]. Recently, soy and lupin proteins have been reported to have hypoglycemic effects.

Most of the protein in lupin seeds (around 44% *w*/*w* of the total conglutins) belongs to the conglutin family, whose molecular mass is between 143 and 260 kDa [82]. β1-, β3-, and β6-conglutin proteins can bind to insulin in vitro, suggesting that β-conglutin proteins have potential benefits for the prevention and treatment of diabetes [83]. The second most prevalent globulin, α-conglutin, shares 33% *w*/*w* of all conglutins. The third conglutin is δ-conglutin, which accounts for 12% *w*/*w* of all conglutins and 4–5% *w*/*w* of all globulins [82]. γ-conglutin (~50 kDa) is a glycoprotein composed of two subunits (17 kDa and 29 kDa). γ-conglutin has excellent hypoglycemic effects in vitro and in vivo (Table 3). In mouse C2C12 cells, γ-conglutin activated the kinases of the synthetic protein pathway and increased glucose consumption by up-regulating muscle-specific gene MHC transcription and GLUT4 gene translocation [73]. In the STZ rat model, Ins1 gene expression and pancreatic insulin content were elevated by γ-conglutin [72]. Bertoglio’s group reported that lupin γ-conglutin had a glucose-lowering effect in a clinical trial [54]. When different dosages of γ-conglutin (630, 315, and 157.5 mg) were eaten 30 min before the carbohydrate supply for 7 weeks, a relevant hypoglycemic effect could be observed in human subjects [54]. Lupin seed protein could be transported by Caco-2 cell monolayers and everted intestinal sacs, suggesting it has the potential to be developed as an oral agent to manage T2D [84].

Soybean storage proteins, such as β-conglycinin and glycinin, also promote anti-diabetic activity (Table 3). Nordentoft reported that soy protein improved insulin sensitivity and up-regulated the expression of key insulin-regulatory genes [69]. A clinical study compared the effects of the intake of 6% (*w*/*v*) whey protein isolate (WPI), whey protein hydrolysate (WPH), soy protein isolate (SPI), and 2.66% WPI, or a control (no protein added) on insulin and glucose responses in 25 healthy men. The results suggested that SPI with 6% protein enhanced insulin response and decreased the plasma glucose level compared with a control beverage [55]. Another study demonstrated that the SPI increased the insulin response compared to WPH [85].

### 3.3. Hydrolytic Peptides

Bioactive hydrolytic peptides are organic substances formed from amino acids and released mainly through enzymatic proteolysis or food processing (cooking, fermentation, ripening) [86,87]. Currently, the commonly used peptide and protein extraction methods mainly include aqueous, organic solvent, enzymatic, ultrasonic, two-phase aqueous, and reverse micelle extraction [86]. The two most common methods for making bioactive peptides are enzyme hydrolysis and fermentation [88]. Pepsin, trypsin, chymotrypsin, alcalase, and flavourzyme are frequently utilized for hydrolysis. Compared with microbial fermentation, these bioactive oligopeptides, occurring in different sizes and produced by enzymes, have higher scalability [4]. The peptides, after enzymatic digestion, can be recovered by desalination, membrane ultrafiltration, column chromatography, and freeze-drying [87].

Crucially, some oligopeptides with short sequences of 2–20 amino acids can be absorbed by the intestine into the blood circulation and exert systemic or local physiological anti-diabetic activities, such as decreasing blood glucose levels, improving glucose and insulin sensitivity in target tissues, and inhibiting key enzymes like α-amylase, α-glucosidase, and DPP-4 related to T2D [4,89,90].

Legume-derived hydrolysates and fractions have also been investigated in cell and animal models (Table 3). Administered with semipurified γ-conglutin, hydrolysates (5 mg/mL) from Andean lupin legumes induced a 6.5-fold glucose uptake and decreased gluconeogenesis in male SD rats by 50% [64]. In Caco-2 cells, treatment with 10 mg/mL black bean hydrolyzed protein isolate (HPI) decreased glucose absorption by 21.5%, and intake of 100–200 mg of HPI/kg BW/day lowered postprandial glucose levels by 22.7–47.7% in a hyperglycemic rat model [60]. The intake of common bean peptide fractions reduced glucose levels in male Wistar rats at the same rate as glibenclamide, keeping a stable basal level without a surge in postprandial hyperglycemia [75]. Jiang found that soybean protein hydrolysate (<5 kDa) made by ultrafiltration significantly reduced the level of fasting blood glucose (FBG) in mice [62]. Wei reported that pea oligopeptides significantly reduced blood glucose levels, lipid profiles, and liver fat deposition in diabetic mice [74].

Some pure sequence-known peptides have also shown hypoglycemic effects in animal and cell models (Table 3). LPYP, IAVPGEVA, and IAVPTGVA from soy glycinin improved glucose metabolism by increasing glucose uptake via up-regulating the expression of GLUT1 and GLUT4 in cultured hepatic cells [63]. Lammi’s group developed a fast, sensitive, and cost-effective ex vivo DPP-4 assay for human serum by collecting venous blood from a healthy female volunteer and analyzing how peptides inactivated the enzyme. They discovered that hydrolytic peptides like LTFPGSAED from lupin β-conglutin and IAVPTGVA from soybean glycinin inhibited the activity of DPP-4 [91].

Furthermore, legume pure peptides and hydrolysates have been found to inhibit key enzymes (α-amylase, α-glucosidase, and DPP-4) in vitro or in silico. Herein, we summarize 40 peptides with clear amino acid sequences and their indicated targets (Table 4). A total of 19 kinds of peptides from the pinto bean, black bean, hard-to-cook bean, chickpea, and fermented bean seeds have been found to inhibit the activities of α-amylases (in biochemical assay or in silico); their sequences, sources, mass weights, and IC50 values are listed in Table 4. In the chemical assay, pinto bean peptides exhibited excellent IC50 values in inhibiting α-amylase. LSSLEMGSLGALFVCM obtained the lowest IC50 value of 0.31 mM compared with others, such as PLPLHMLP (5.92 mM), PPMHLP (6.08 mM), PPHMGGP (6.14 mM), PLPWGAGF (6.64 mM), and PPHMLP (23.33 mM) [92,93]. Some α-amylase inhibitory peptides were identified in fermented bean seeds, such as INEGSLLLPH FVVAEQAGNEEGFE, SGGGGGGVAGAATASR, GSGGGGGGGFGGPRR, GGYQGGGYGGNSGGGYGNRG, GGSGGGGGSSSGRRP, and GDTVTVEFDTFLSR. Their IC50 values ranged from 0.04 μg/mL to 0.65 μg/mL [94]. In in silico study, some peptides seemed to provide potent inhibition against α-amylase, α-glucosidase, and DPP-4 by computational modelling. Compared with acarbose (−9.71 kcal/mol), a known α-amylase inhibitor, the free energy (interaction with the catalytic site of α-amylase) of peptides AKSPLF and WEVM were −10.2 kcal/mol and −10.1 kcal/mol, respectively [95]. QQEG from hard-to-cook beans inhibited α-amylase, and the interaction score was −7.29 REU [66]. Twelve peptides provided the potential inhibition of α-glucosidase, including aglycin, LLPLPVLK, SWLRL, WLRL, GSR, EAK, TTGGKGGK, KKSSG, GGGLHK, CPGNK, KTYGL, and YVDGSGTPLT (Table 4). The α-glucosidase inhibitory activity of aglycin from soybeans was 36.48 μmol/L [96]. GSR and EAK, identified from soybeans, had IC50 values of 20.40 µM and 520.20 µM, respectively [62]. LLPLPVLK, SWLRL, and WLRL from soy protein showed strong α-glucosidase inhibitory activity, with IC50 values of 237.43 ± 0.52 µM, 182.05 ± 0.74 µM, and 162.29 ± 0.74 µM, respectively [97]. Four synthesized peptides (KKSSG, GGGLHK, CPGNK and KTYGL) were evaluated with the α-glucosidase inhibitory ratio of 49.34 ± 6.5%, 46.10 ± 8.30%, 37.60 ± 6.8%, and 36.30 ± 8.80%, respectively [98].

Peptides from the lupin bean, soybean, fermented soybean, black bean, common bean, chickpea, and hard-to-cook bean exhibited inhibition of DPP-4. Peptides LTFPGSAED, IAVPTGVA, EGLELLLLLLAG, AKSPLF, FEELN, KKSSG, GGGLHK, CPGNK, KTYGL, KL, and LR were confirmed to inhibit the activity of DPP-4 by biochemical assay. RGPLVNPDPKPFL, PHPATSGGGL, YVDGSGTPLT, LLSL, and QQEG were identified as potential DPP-4 inhibitors by computational modelling (Table 4).

In addition, several studies showed that a hydrolysate and peptide fraction mixture had the potential to inhibit the enzymatic activities of α-amylase, α-glucosidase, and DPP-4 (Table S1). Alkaline protease was reported to be the best enzyme for producing black bean protein components with anti-diabetic potential [60,95]. Inhibition values for DPP-4, α-amylase, and α-glucosidase of alcalase fraction peptides from the black bean were 96.70%, 53.40%, and 66.10%, respectively [95]. The inhibition of α-glucosidase in different common bean cultivars’ protein hydrolysates ranged from 46.90–60.00% [101], and the IC50 of DPP-4 inhibition ranged from 0.14–0.33 mg DW/mL [98]. In another in vitro study, 5 mg/mL purified γ-conglutin hydrolysates from lupin inhibited DPP-4 completely. The fact that undigested γ-conglutin did not directly inhibit DPP-4 activity demonstrated that this protein needs to be digested to produce DPP-4 enzyme inhibitory hydrolysates [64]. Bambara bean hydrolysates produced by alcalase and thermolysin exhibited similar DPP-4 inhibitory activity with an IC50 of 1.73 mg/mL [102]. Cowpea alcalase protein hydrolysis also showed strong DPP-4 inhibition [103,104]. Soybean hydrolysates had an α-glucosidase inhibitory activity with an IC50 of 0.05 mg/mL [62]. Six-days-germinated soybean protein hydrolysates inhibited DPP-4 (IC50 = 1.49 ± 0.14 mg/mL), α-amylase (IC50 = 1.70 ± 0.18 mg/mL), and sucrase activities of α-glucosidases (IC50 = 2.90 ± 0.07 mg/mL). Peptide fractions of 5–10 kDa and >10 kDa were more effective at inhibiting DPP-4 (IC50 = 0.91± 0.17 mg/mL and 1.18 ± 0.15 mg/mL, respectively) [61].

Collectively, legume peptides and hydrolysates show remarkable hypoglycemic effects through various approaches. Interest has been directed toward the acceptable oral administration of bioactive peptides, one of the main attractions for T2D patients. Based on their inherent amino acid composition and sequence, bioactive peptides exhibit different activities in regulating glucose and lipid metabolism, which exert beneficial effects on the control of diabetic progress.

## 4. Potential Hypoglycemic Mechanism of Bioactive Legume Peptides

**Figure 1:**
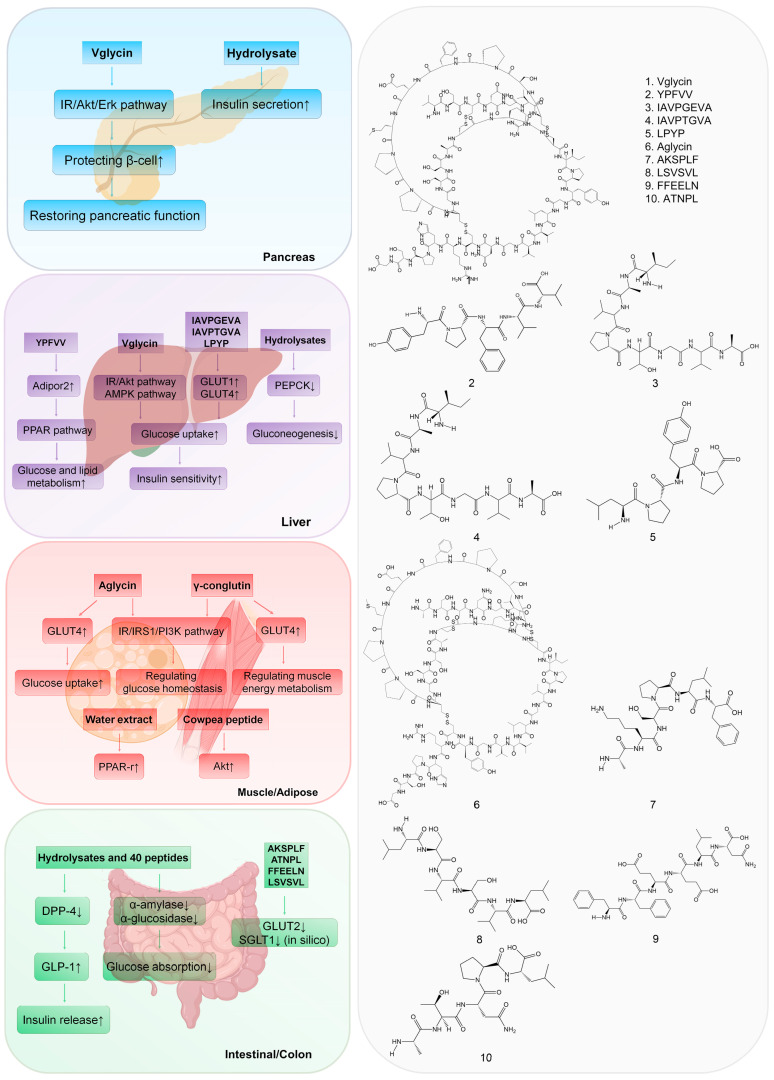
Mechanism of anti-diabetic effects of 10 representative legume-derived peptides. The amino acid sequence or molecular structure of the peptides are presented in the (**right**) panel. The anti-diabetic mechanisms of these peptides in corresponding organs are showed in the (**left**) panel. ↑ means a stimulative effect and ↓ means an inhibitory effect in the text. Peptide 1 works in pancreas; peptides 1 to 5 work in liver; peptide 6 works in muscle and adipose cells; and peptides 7–10 work in intestinal/colon tissue.

The hypoglycemic mechanism of legume diabetic peptides mainly includes reducing glucose absorption, promoting pancreatic β-cells proliferation, enhancing insulin secretion and sensitivity through signaling pathways associated with diabetes, and inhibiting carbohydrate-digesting enzymes (α-amylase and α-glucosidase) and DPP-4 in target organs (Figure 1).

### 4.1. Targeting the Pancreas

One of the main factors contributing to the development of T2D is assumed to be a decrease in glucose-stimulated insulin secretion from pancreatic β-cells [105]. An appealing idea for treating diabetes and its complications is to restore the function of poor β-cells. The IRS (insulin receptor substrate)/AKT (protein kinase B) pathway is the primary mechanism of the hypoglycemic effect of the body’s response to insulin stimulation [106]. Vglycin peptide exhibited beneficial effects on hyperglycemia by facilitating glucose metabolism and enhancing insulin sensitivity, but it could not increase insulin secretion [57]. Another study supports an apparent impact of vglycin on regulating β-cell preservation; a self-renew program was initiated in which vglycin directly promoted the proliferation of β-cells via activating the IR/Akt/Erk pathway [14]. These results reveal the protective effects of vglycin on improving β-cell function in long-term glucolipotoxicity.

Some hydrolysates and fractions also exhibited a protective effect on the dysfunctional pancreas. Hydrolysates (<3 kDa) from hard-to-cook beans and common beans were reported to increase glucose-stimulated insulin secretion up to 57% and 45% from the basal state in INS-1E pancreatic β-cells, respectively [66,76]. In another study, fermented meju water extracts (W-60), containing 15 kDa peptides, increased glucose-stimulated insulin secretion capacity and β-cell viability in Min6 insulinoma cells [65]. Previous studies have identified that amino acid metabolism, especially glutamine and alanine, is essential for the functional maintenance of pancreatic cells and insulin secretion [107]. Therefore, it can be inferred that the amino acids or oligopeptides in the hydrolysates of legumes may be beneficial for the exertion of hypoglycemic function of the pancreas directly or indirectly.

### 4.2. Targeting the Liver

Soymorphin-5 (YPFVV), derived from the soy conglycinin subunit, plays a role in glucose and lipid metabolism [58]. Although no differences were observed in the weights of mesenteric and epididymal fat and brown adipose tissues, plasma TG levels and liver weights in KKA^y^ mice were significantly reduced by soymorphin-5 treatment, suggesting that soymorphin-5 enhanced the liver lipid metabolism. In terms of mechanism, soymorphin-5 improved fatty acid β-oxidation and energy expenditure by increasing the hepatic expression of PPAR and its target genes, including Adipor2, AOX1, CPT1, and UCP2 [58]. In another study, vglycin was probably responsible for increased fatty acid β-oxidation via the AMPK pathway and inhibited fatty acid synthesis (FAS) by down-regulating expression of FAS in HFD-fed C57BL/6J mice [15]. Soy peptides IAVPGEVA, IAVPTGVA, and LPYP enhanced glucose uptake through GLUT1 and GLUT4 activation, and modulated glucose metabolism by activating Akt and AMPK pathways in HepG2 cells [63]. In addition, γ-conglutin hydrolysates decreased gluconeogenesis by 50% in a dual-layered enterocyte/hepatocyte system and down-regulated the expression of phosphoenolpyruvate carboxykinase (PEPCK) in HepG2 cells [64].

### 4.3. Targeting Muscle and Adipose Tissue

Increasing glucose absorption and transformation in muscle and adipose tissue is an effective method to control hyperglycemia. As shown in a STZ/HFD-induced diabetic mice model, oral administration of aglycin could potentially attenuate or prevent hyperglycemia via enhancing the IR/IRS1 signaling pathway in skeletal muscle [56]. Additionally, in C2C12 cells, aglycin increased glucose uptake by recruiting glucose transporter GLUT4 to the surface of cells [56]. Cowpea peptides induced the Akt phosphorylation in skeletal cells and mimicked the actions of insulin by activating the same signaling pathway [67]. Prior findings strongly implied that the translocation of GLUT4 was regulated by activation of the Akt signaling pathway in the skeletal muscle cells, which facilitated glucose uptake [108,109].

Studies of fermented soy products have suggested that the small bioactive peptides produced during fermentation may contribute to preventing or slowing the progression of T2D [65,110]. Water extracts (<3 kDa) from chungkookjang increased PPAR-r activity by about 59%, indicating that there exist certain small peptides that work as mild PPAR-r agonists in glucose and lipid metabolism [65]. γ-conglutin hydrolysates also significantly increased the insulin-induced glucose uptake in mature 3T3-L1 adipocytes through an enhanced expression of GLUT4, similar to metformin treatment [64]. Similarly, in C2C12 cells, γ-conglutin increased flottilin-2, caveolin-3 concentrations, and CBL phosphorylation, resulting in GLUT4 translocated on the cell membrane, which suggested that γ-conglutin plays an essential role in muscle energy metabolism through insulin-mimetic action [73].

### 4.4. Targeting the Intestine and Colon

In STZ-induced Wistar rats and the Caco-2 cell models, black bean peptides AKSPLF, ATNPL, FFEELN, and LSVSVL may have reduced glucose absorption via blocking glucose transporters GLUT2 and SGLT1 (Figure 1) [60]. In Table 4, we summarized 19 α-amylase inhibitors, 12 α-glucosidase inhibitors, and 16 DPP-4 inhibitors from legume peptides, all of which worked as potential anti-diabetic agents targeting the intestine and colon (Figure 1). The mechanism of lowering postprandial hyperglycemia by inhibition of α-amylase and α-glucosidase is to inhibit the rate of glucose release and absorption in the intestine [12]. The hypoglycemic mechanism of inhibiting the DPP-4 enzyme is to retard the decomposition of GLP-1 and increase the endogenous physiological level of GLP-1 in the human body [34]. Recently, the structure of oligopeptides and their binding modes to these enzymes have been systematically investigated, which may provide a clear anti-diabetic mechanism for these peptides [40].

α-amylase contains three distinct structural domains (A, B, and C). The active site region in domain A is associated with an extended substrate binding cleft, allowing it to recognize polymeric starch molecules [111]. Asp197, Glu233, and Asp300 are three essential catalytic carboxylic acid residues located within this active site cleft between the carboxyl ends of domains A and B. Domain B is responsible for substrate specificity and enzyme stability, and domain C helps to maintain the stability of the catalytic site. Therefore, blocking the catalytic sites was an effective way to inhibit the activity of α-amylase [40]. Peptides amply containing aromatic residues such as Leu, Pro, Gly, and Phe were effective inhibitors of α-amylase because the substrate-binding pockets of the α-amylase enzyme have many aromatic residues [111]. Hence, apart from interactions involving hydrogen bonds and electrostatic and van der Waals interactions, aromatic–aromatic interactions have also played a crucial role in α-amylase inhibitory activity [4].

The bioactive peptides LSSLEMGSLGALFVCM, PLPLHMLP, PPMHLP, PPHMLP, PPHMGGP, and PLPWGAGF, extracted from pinto beans, interacted or bound to the indicated domain of α-amylase, ultimately restricting protein conformation and affecting the degree of enclosure of the substrate. Mechanically, when the (α-amylase)–starch complex was formed, the uncompetitive peptide inhibitors bound to the complex, resulting in the starch no longer being able to hold on at its original position due to the altered conformation of α-amylase. Consequently, the starch was detached from the (α-amylase)–starch complex [92]. The competitive inhibitors GSR and EAK formed hydrogen bonds with the catalytic residues [62].

Structural analysis has clearly shown the intimate interaction between bean-derived peptides and α-amylase catalytic residues. The interaction prevented residue Asp300 from adopting its functional position and destroyed the water channel leading from the “flexible loop” to the heart of the active-site depression [112]. Another inhibitory mechanism was identified in the interaction of porcine pancreatic α-amylase and the bean P. vulgaris-derived peptide, in which residue His305 participated in substrate binding and structural changes [113].

Similar to peptides that inhibit α-amylase, certain peptides have the potential to inhibit α-glucosidase, reducing glucose absorption and delaying carbohydrate digestion, thereby reducing postprandial blood glucose levels. Pure peptides such as KTYGL were able to inhibit the enzyme mainly through polar interactions (Asn32, Asp34, Asp38, and Asp89), hydrophobic interactions (Trp36 and Trp81), and hydrogen bonds (Ser31) with the residues from the binding pocket of the α-glucosidase [98]. AKSPLF showed only hydrogen bonding and polar interactions with α-glucosidase, and Ala and Lys were the only two amino acids interacting with ASP34, THR83, ASP89 and ASN32 [95]. Besides, α-glucosidase inhibitors also played crucial roles in the secretion of GLP-1 in T2D [114].

DPP-4 is a serine exopeptidase that cleaves X-proline or X-alanine dipeptides from the N-terminus of polypeptides, which degrades and inactivates GLP-1 and GIP. Several legume-derived peptides have been identified as promising DPP-4 inhibitors, potentially contributing to glycemic control. DPP-4 has hydrophobic pockets, which are crucial targets for inhibition of the enzyme [115]. Generally, the DPP-4 inhibitory peptides often contain high concentrations of hydrophobic amino acids such as alanine, glycine, isoleucine, leucine, phenylalanine, proline, methionine, tryptophan, and valine [4]. The interactions between DPP-4 and common bean peptides (KTYGL and AKSPLF) were mainly H-bond, hydrophobic, polar and cation π bonds [85,92]. The interactions of DPP-4 with peptide AKSPLF occurred primarily between amino acids GLU191, ASP192, ARG253, and LEU235 [95].

Collectively, legume peptides exhibit great inhibitory potential due to their affinity for and specificity of action on key enzymes such as α-amylase, α-glucosidase, and DPP-4, which are promising candidates for the development of anti-diabetic drugs. However, the structure–activity relationships between peptides and these enzymes remain to be uncovered.

## 5. Challenges of Legume Peptides’ Development in T2D Treatment

Despite their tremendous potential value in anti-T2D therapies, legume-derived peptides have intrinsic disadvantages, including poor chemical and physical stability, poor bioavailability, short effective half-life, and inefficiency of oral delivery, which must be considered for their application as nutraceuticals or medicines [12,116]. Effectively delivering therapeutic peptides via the oral route is still a big challenge, even though some drawbacks can be solved by rational design with the increase of biological activity, selectivity, or bioavailability. Numerous studies have concentrated on creating novel technologies through physical and chemical methods, including enteric coating, enzyme inhibitors, permeability enhancers, nanoparticles, and intestinal microdevices, to improve the oral bioavailability and stability of the peptides, which overcome the absorptive barriers in the gastrointestinal tract [4,22,117].

Low selectivity, resistance to protein hydrolysis, and the demands of large-scale production are also some of the main challenges [22]. Approaches such as proteomic analysis, bioinformatics tools, pretreatments, hydrolysis mechanisms and isolation, and effective cell and animal screening models all contribute to obtaining hydrolysates or peptides with hypoglycemic activity [118]. Regarding the delivery and administration of peptides in the human body, the effectivity and stability of the peptides in the gastrointestinal tract are worth considering. Since oligopeptides are predicted to be resistant to digestive enzymes, they can be utilized as an ingredient or supplement in nutraceutical foods. Those specific peptides with long amino acid sequences or hydrolysates are still susceptible to digestive enzymes.

In addition, some legume-derived anti-diabetic peptides are mainly studied by integrated computational methods or in vitro assays, due to a lack of sufficient animal and human clinical trials. These potential anti-diabetic peptides need a deep investigation geared towards the development of nutraceuticals and drugs. On the other hand, hydrolytic peptides are generated from dietary proteins during gastrointestinal digestion or are produced by processing proteins. Their bioavailability after oral administration depends on their absorption and distribution to reach target organs [119]. The peptides in the legume hydrolysates (Table S1) should be identified, and their anti-diabetic activities and precise mechanisms need further investigation. Undoubtedly, legume-derived peptides research can benefit from pharmaceutical approaches for the discovery, design, and development of nutraceutical or oral medicinal peptides for T2D.

## 6. Conclusions and Perspectives

Peptides are becoming one of the most attractive candidates for the development of oral anti-diabetic drugs. The FDA has approved Rybelsus^®^ (semaglutide) oral tablets to improve control of blood sugar in adult patients with T2D, along with diet, exercise, and other anti-diabetic drugs. The efficacy and safety of the treatment of Rybelsus^®^, assisted with Salcaprozate sodium, an absorption enhancer, have been considered acceptable in several clinical trials [31,120,121]. It provides a viable design for oral legume-derived drug development for T2D. The biopeptides and protein hydrolysates from legumes summarized in this review are confirmed to have anti-diabetic activities, and their anti-diabetic mechanisms are illustrated in vivo and in vitro.

The biological activity of a peptide mainly depends on its chemical structure, which determines its molecular function in target organs [44,94]. Structural modifications can enhance the enzymatic tolerance of polypeptide drugs, thereby increasing the half-life of drugs. As a native peptide, aglycin consists of 37 amino acids and contains the special cystine knot structure in the molecule, which can resist the hydrolysis of proteases in the digestive tract. This self-protective structural feature makes aglycin viable as an oral agent in the management of T2D and other metabolic syndromes.

Since its discovery, insulin has been one of the major therapeutic molecules used to control diabetes. The most common route of insulin administration is the subcutaneous route. However, oral delivery is the most convenient and patient-centered route. The human physiological system acts as a formidable barrier to insulin, limiting its bioavailability. Currently, the delivery methods enhancing the bioavailability of oral insulin and GLP-1 receptor agonists include the use of ionic liquids, hydrogels, nanoparticles, microparticles, and nano-in-microparticles [122]. These strategies provide new insights into the development of the effective delivery of orally administered protein and peptide drugs.

## Figures and Tables

**Table 1:** Clinical drugs in T2D and their target organs/tissues.

Target Organs/Tissues	Mechanism	Drug Class	Drug Name	Adverse Reaction	Drug Delivery
Pancreatic β-cell	β-cell mass restore, increasing insulin secretion	Sulfonylurea	Glibenclamide, glipizide, glimepiride, gliclazide	Cause hypoglycemia	Oral
		Meglitinide	Repaglinide, nateglinide	Not cause hypoglycemia	Oral
Pancreatic α-cell	Increasing glucagon secretion	GLP-1 agonist	Exenatide, lixisenatide, liraglutide, dulaglutide, exenatide extend-release, semaglutide, albiglutide	Gastrointestinal and hypoglycemic reactions	Inject/Oral
		Amylin analog	Pramlintide	Cause hypoglycemic	Inject
		Biguanide	Metformin	Lactic acidemia and ketouria	Oral
Liver	Decreasing hepatic glucose production	Thiazolidinedione	Pioglitazone, rosiglitazone	Weight gain, edema, cardiac problems	Oral
		Insulin	Rapid-, short-, intermediate-, long-acting insulin	Hypoglycemia and edema	Inject
		Biguanide	Metformin	Lactic acidemia and ketouria	Oral
Muscle and adipose tissue	Increasing glucose uptake and glycolysis	Biguanide	Metformin	Lactic acidemia and ketouria	Oral
		Thiazolidinedione	Pioglitazone, rosiglitazone	Weight gain, edema, cardiac problems	Oral
		Insulin	Rapid-, short-, intermediate-, long-acting insulin	Hypoglycemia and edema	Inject
		α-glucosidase inhibitor	Acarbose, miglitol, voglibose	Gastrointestinal reaction	Oral
Intestine	Increasing incretin activity, enhancing glucose absorption	DPP-4 inhibitor	Sitagliptin, saxagliptin, linagliptin, vidaglipti, alogliptin, teneligliptin, gemigliptin	Minor adverse drug reactions	Oral
		GLP-1 agonist	Exenatide, lixisenatide, liraglutide, dulaglutide, exenatide extend-release, semaglutide, albiglutide	Gastrointestinal and hypoglycemic reactions	Inject/Oral
		Biguanide	Metformin	Lactic acidemia and ketouria	Oral
Colon	Balancing gut microbiota	GLP-1 agonist	Exenatide, lixisenatide, liraglutide, dulaglutide, exenatide extend-release, semaglutide, albiglutide	Gastrointestinal and hypoglycemic reactions	Inject/Oral
Kidney	Inhibiting glucose reabsorption	SGLT-2 inhibitor	Dapagliflozin, canagliflozin, empagliflozin, ertugliflozin, Sotagliflozin	Urinary genital infection risk, ketoacidosis risk	Oral
Central nervous system	Lowing neurotransmitters	Dopamine-receptor agonist	Bromocriptine	Gastrointestinal reaction	Oral

**Table 2:** Risk of T2D in relation to the average intake of legumes and their proteins.

Source	Consumption(g/d)	People Tested	HRs or RR (95% CIs)	Reference
Lentils	6.6	Caucasian	0.67 (0.46, 0.98)	[47]
Chickpea	5.0	Caucasian	0.68 (0.46, 1.00)	[47]
Soybean	32.0	Chinese women	0.57 (0.48, 0.60)	[48]
Soy protein	11.0−15.0	Chinese/Japanese	0.84 (0.75, 0.95)	[49]
Soy protein	13.6	Japanese women	0.46 (0.30, 0.70)	[50]

CIs, confidence intervals; HRs, hazard ratios; RR, relative risk.

**Table 3:** Hypoglycemic effect and mechanism of legume peptides and hydrolysates in vivo and in vitro.

Peptide	Amino Acid Sequence	Source	Model	Feeding/Treat Pattern	Hypoglycemic Effect and Index	Mechanism	Reference
Aglycin	ASCNGVCSPFEMPPCGSSACRCIPVGLVVGYCRHPSG	Soybean	STZ/HFD-induced diabetic BALB/c mice, C2C12 cell	50 mg/kg/d, 4 weeks	BG↓, OGTT↑, insulin tolerance↑, p-IR↑, p-IRS1↑, p-Akt↑, GLUT4↑, glucose uptake↑	Increasing insulin receptor via IR/IRS1 pathway, enhancing glucose uptake	[56]
Vglycin	VSCNGVCSPFEMPPCGSSACRCIPYGLVVGNCRHPSG	Pea seed/germinating pea seed	STZ/HFD-induced diabetic rats, HepG2 cell, L02 cell	4 weeks	BW↓, food intake↓, FPG↑, PARP↑, PDX1↑, GSK3α/β↑, GLUT4↑, p85-PI3-kinase↓, p-Akt↓	Impairing glucose tolerance, restoring pancreatic function, enhancing insulin signaling by activating the IR/Akt pathway	[57]
			T1D SD rat, STZ-induced T2D C57BL/6 mice, INS-1 832/13 cell	80 mg/kg/d, 4 weeks, 8 weeks	BW↓, lee’s index↓, food intake↓, FPG↓, BG↓, ITT↑, pancreatic islet↑, plasma insulin↑, glucose-stimulated glucagon↓, pancreatic mass↑, β-cell area and mass↑, Ki67/PCNA immunostaining area↑, TUNEL-positive/total β-cells↓, Erk↑	Promoting the proliferation of β-cells via the IR/Akt/Erk pathway	[14]
			HFD-C57BL/6J mice, HepG2 cell	15 weeks	BW↓, FPG↓, glucose tolerance↑, ITT↑, FA β-oxidation↑, FAS↓	Improving insulin sensitivity and glucose tolerance, enhancing β-oxidation and inhibiting FAS via AMPK pathway and down-regulating the FAS	[15]
Soymorphin-5	YPFVV	Soybean	KKA^y^ mice	10 mg/kg/d, 5 weeks	BG↓, plasma insulin↓, TG↓, adiponectin↑, liver TG↓, Adipor2↑, PPARα↑, AOX1↑, CPT1↑, UCP2 1↑	Increasing insulin sensitivity, improving glucose and lipid metabolism via activation of the adiponectin and PPARα system	[58]
Soy protein	/	Soybean	ZDF rat	Soy protein, 11 weeks	BW↓, total adiposity↓, total and liver adiposity↓, glucose level↓, insulin↓, GLUT4↑, PPAR-r↑, FAS↓, GPDH↓	Maintenance of peripheral (adipose tissue) insulin signaling	[68]
Soy protein	/	Soybean	KKA^y^ mice	High content isoflavone soy protein, 9 weeks	FPG↓, insulin↓, TG↓, TC↓, GLUT2↑, GLUT3↑, Ins1↑, Ins2↑, IGF1↓, β2/Neurod1↓, cholecystokinin↓, LDLR↑	Improving glucose and insulin sensitivity	[69]
γ-conglutin	/	Lupin bean	SD rat, HepG2 cell	10 μmol/L, 24 or 48 h	Glucose consumption↑	Maintaining glucose homeostasis	[59]
γ-conglutin	/	Lupin bean	STZ-induced Wistar rat	150 mg/kg, 1 week	G6pc↓, Fbp1↓, Pck1↓	Regulating glucose metabolism mainly through G6pc inhibition	[70]
γ-conglutin	/	Lupin bean	Glucose-administrated male rat	50, 100 and 200 mg/kg BW, 0, 30, 60 and 90 min	BG↓, AUC↓	Hypoglycemic effect	[54]
γ-conglutin	/	Lupin bean	Glucose-administrated male rat	30, 60 and 120 mg/kg BW, 30, 60, 90, and 120 min	Plasma glucose↓	Hypoglycemic effect	[71]
γ-conglutin	/	Lupin bean	STZ administrated rat	120 mg/kg in saline, 1 week	Glucose↓, Ins1↑, pancreatic insulin↑, serum insulin↑	Hypoglycemic effect	[72]
γ-conglutin	/	Lupin bean	C2C12 cell	0.5 mg/mL,72 h	Plasma glucose↓, IRS1 protein↑, p85-PI3 kinase↑, Akt1 protein↑, eIF4E protein↑, p70S6 K↑, ERK1↑, ERK2↑, MHC protein↑, myogenin↑	Regulating muscle energy metabolism, protein synthesis and MHC gene transcription through insulin signaling pathway	[73]
HPI/pure peptide	AKSPLF, ATNPL, FFEELN, LSVSVL	Black bean	STZ-induced Wistar rats, Caco-2 cell	100, 150 and 200 mg HPI/kg/BW, 15 d, pure peptide (100 µM), HPI (10 mg/mL)	BW↓, BG↓, postprandial glucose level↓, insulin↓, GLP-1↑, OGTT↓, glucose absorption↓, GLUT2↓, SGLT1↓	Reducing glucose absorption via blocking glucose transporters GLUT2 and SGLT1(in silico)	[60]
Synthetic peptide	IAVPGEVA, IAVPTGVA, LPYP	Soy glycinin	HepG2 cell	50 and 100 µM	Glucose uptake↑, Akt↑, GSK3α/β↓, GLUT4↑, GLUT1↑	Enhancing glucose uptake through GLUT1 and GLUT4 activation, Akt and AMPK pathway	[63]
Hydrolysate	/	Soy protein	Alloxan-induced male Kunming mice	47.5 mg/kg/d, 3 weeks	FBG↓	Hypoglycemic effect	[62]
Hydrolytic oligopeptide	/	Pea	STZ/HFD-induced diabetic mice	800, 1600 and 3200 mg/kg BW, 4 weeks	FBG↓, BW↓, OGTT↑, serum insulin↑, TC↓, TG↓, HDL-C↑, fatty acid anion ↓, liver and muscle glycogen↑	Enhancing insulin sensitivity	[74]
Protein/peptide fraction	<10 kDa	Common bean	Male Wistar rat	0.5 and 5 mg/kg	Glucose level↓, glucose uptake↑	Hypoglycemic activity	[75]
γ-conglutin hydrolysate	/	Lupin bean	Caco-2 cells, 3T3-L1 cell, HepG2 cell	2 and 5 mg/mL	Glucose uptake↑, gluconeogenesis↓, PEPCK↓, GLUT4↑, HepG2 glucose production↓	Inhibiting DPP-4, improving insulin receptor sensitivity, inhibiting hepatic gluconeogenesis through GLUT4 activation	[64]
Peptide	/	Cowpea	Rat L6 skeletal muscle cell	Various doses	Akt↑	Activating the insulin signaling pathway	[67]
Water extract peptide	<3 kDa	Fermented soybean	3T3-L1 cell, Min6 cells NCI-H716 cell, Human embryo kidney 293 cell	5 µg/mL	Glucose uptake↑, triacylglycerol↑, PPAR-r↑, β-cell viability↑, insulin secretion↑, cell proliferation↑, PDX1↑	Increasing insulin sensitivity and exerting insulinotropic via PPAR-r activation	[65]
Hydrolysate/ fraction	<1 and 1–3 kDa	Hard-to-cook bean	INS-1E cell	1 g Hydrolysate treatment	Increase insulin secretion up to 57%	Increasing insulin secretion	[66]
Hydrolysate	/	Common Bean(G0-0h)	INS-1E pancreatic β-cell	2 mg SP/mL	Increase insulin secretion 45% from the basal state	Increasing insulin secretion	[76]

G0-0h, the non-germinated and non-hydrolyzed sample; HPI, hydrolyzed protein isolate; SP, soluble protein; ZDF, Zucker diabetic fatty; ↑stimulative effect; ↓inhibitory effect.

**Table 4:** Legume-derived peptide inhibitors targeting α-amylase, α-glucosidase, and DPP-4.

NO.	Peptide Sequence	MW(DA)	Source	Target	IC50, %	Reference
1	LSSLEMGSLGALFVCM	1658.02	Pinto bean	α-amylase	0.31 mM	[92]
2	PLPLHMLP	917.18	Pinto bean	α-amylase	5.92 mM	[92]
3	PPMHLP	690.87	Pinto bean	α-amylase	6.08 mM	[92]
4	PPHMLP	690.81	Pinto bean	α-amylase	23.33 ± 0.15 mM	[93]
5	PPHMGGP	691.81	Pinto bean	α-amylase	6.14 mM	[92]
6	PLPWGAGF	843.98	Pinto bean	α-amylase	6.64 mM	[92]
7	SPQSPPFATPLW	1327.50	Chickpea	α-amylase	−8.40 kcal/mol	[99]
8	FVVAEQAGNEEGFE	1525.59	Fermented bean seed	α-amylase	0.04−0.65 μg/mL	[94]
9	SGGGGGGVAGAATASR	1232.29	Fermented bean seed	α-amylase	0.59−2.12 μg/mL	[94]
10	GSGGGGGGGFGGPRR	1232.29	Fermented bean seed	α-amylase	0.59−2.12 μg/mL	[94]
11	INEGSLLLPH	1092.26	Fermented bean seed	α-amylase	0.04−0.65 μg/mL	[94]
12	GGYQGGGYGGNSGGGYGNRG	1791.77	Fermented bean seed	α-amylase	0.59−2.12 μg/mL	[94]
13	GGSGGGGGSSSGRRP	1232.24	Fermented bean seed	α-amylase	0.59−2.12 μg/mL	[94]
14	GDTVTVEFDTFLSR	1586.72	Fermented bean seed	α-amylase	0.59−2.12 μg/mL	[94]
15	NEGEAH	655.62	Hard-to-cook bean	α-amylase	−12.84 REU	[66]
16	FFL	425.53	Hard-to-cook bean	α-amylase	−8.22 REU	[66]
17	WEVM	563.67	Black bean	α-amylase	0.04 µM (ki)	[95]
18	AKSPLF	661.79	Black bean	α-amylase, DPP-4	0.03 µM (ki), 0.08 µM (ki)	[95]
19	QQEG	460.44	Hard-to-cook bean	α-amylase, DPP-4	−7.03 REU, −7.29 REU	[66]
20	Aglycin ^1^	3743.40	Soybean, pea	α-glucosidase	36.48 μM	[96]
21	LLPLPVLK	892.18	Soybean	α-glucosidase	237.43 ± 0.52 µM	[97]
22	SWLRL	673.80	Soybean	α-glucosidase	182.05 ± 0.74 µM	[97]
23	WLRL	586.73	Soybean	α-glucosidase	162.29 ± 0.74 µM	[97]
24	GSR	318.33	Soybean	α-glucosidase	20.40 µM	[62]
25	EAK	346.38	Soybean	α-glucosidase	520.20 µM	[62]
26	TTGGKGGK	704.77	Black bean	α-glucosidase	0.27 µM (ki)	[95]
27	KKSSG	505.57	Common bean	α-glucosidase, DPP-4	49.34 ± 6.5%, 0.64 ± 0.16 mg/mL	[98]
28	GGGLHK	567.65	Common bean	α-glucosidase, DPP-4	46.10 ± 8.30%, 0.61 ± 0.10 mg/mL	[98]
29	CPGNK	517.60	Common bean	α-glucosidase, DPP-4	37.60 ± 6.8%, 0.87 ± 0.02 mg/mL	[98]
30	KTYGL	580.68	Common bean	α-glucosidase, DPP-4	36.30 ± 8.80%, 0.03 mg/mL	[98]
31	YVDGSGTPLT	1009.08	Chickpea	α-glucosidase, DPP-4	−7.30 kcal/mol, −8.20 kcal/mol	[99]
32	IAVPTGVA	726.87	Soybean	DPP-4	223.20 µM (in situ)	[91]
33	LTFPGSAED	935.97	Lupin bean	DPP-4	207.50 µM (in situ)	[91]
34	EGLELLLLLLAG	1253.52	Black bean	DPP-4	0.06 µM (ki)	[95]
35	FEELN	650.69	Black bean	DPP-4	0.10 µM (ki)	[95]
36	RGPLVNPDPKPFL	1449.72	Common bean	DPP-4	14.04 kcal/mol	[76]
37	KL	259.34	Fermented soybean	DPP-4	41.40 ± 2.68 µg/mL	[100]
38	LR	287.35	Fermented soybean	DPP-4	598.02 ± 18.35 µg/mL	[100]
39	PHPATSGGGL	892.97	Chickpea	DPP-4	−8.20 kcal/mol	[99]
40	LLSL	444.57	Hard-to-cook bean	DPP-4	−11.75 REU	[66]

^1^ The amino acid sequence of aglycin is in Table 3. Amino acid nomenclature: C, cysteine; H, histidine; I, isoleucine; M, methionine; S, serine; V, valine; A, alanine; G, glycine; L, leucine; P, proline; T, threonine; F, phenylalanine; R, arginine; Y, tyrosine, W, tryptophan; D, aspartic acid; N, asparagine; E, glutamic acid, Q, glutamine; K, lysine. Ki, inhibition constant expressed in µM.

## Data Availability

Not applicable.

## Supplementary Materials

The following supporting information can be downloaded at: https://www.mdpi.com/article/10.3390/nu15051096/s1, Table S1: Hydrolytic fractions from legume targeting α-amylase, α-glucosidase, and DPP-4.

## Abbreviations

Akt	protein kinase B
AUC	area under curve
BG	blood glucose
BW	body weight
Cγ	γ-conglutin
DPP-4	dipeptidyl peptidase IV
FA	fatty acid
FAS	fatty acid synthase
FBG	fasting blood glucose
FPG	fasting plasma glucose
GIP	gastric inhibitory peptide
GLP-1	glucagon-like peptide-1
GLUT4	glucose transporter type 4
HFD	high-fat diet
IC50	half maximal inhibitory concentration
IR	insulin receptor
IRS1	insulin receptor substrate 1
ITT	insulin tolerance test
OGTT	oral glucose tolerance test
PPAR	peroxisome proliferator-activated receptor
REU	rosetta energy units
SD	Sprague Dawley
SGLT-2	sodium-glucose cotransporter-2
STZ	streptozotocin
T2D	type 2 diabetes
TG	triglyceride
TZDs	thiazolidinediones
