# Supplementary material for: Legume-Derived Bioactive Peptides in Type 2 Diabetes: Opportunities and Challenges
Source: Nutrients. 2023 Feb 22;15(5):1096. doi: 10.3390/nu15051096 (PMC10005352; doi:10.3390/nu15051096)
Supplement: Supplementary file 1 [file nutrients-15-01096-s001.zip › nutrients-2236327-supplementary.pdf]

Table S1 Hydrolytic fractions from legume targeting α-amylase, α-glucosidase and DPP-4

Source	Fraction	MW(KDa)	Inhibition index (IC50, %)			Reference
			α-amylase	α-glucosidase	DPP-4	
Pinto bean	Protamex fraction	< 3.0	62.10 ± 3.49%			[111]
	Bromelain fraction	< 1.0	49.90 ± 1.40%			[66]
	Alcalase fraction	< 1.0	/	76.40 ± 0.50%	55.30 ± 1.60%	
Cowpea	Alcalase fraction	> 10.0	31.58 mg/mL	0.63 mg/mL		[104]
	Pepsin-pancreatin fraction	< 1.0	40.17 mg/mL	189.04 mg/mL		
	Alcalase hydrolysate	/			0.58 mg SP/mL	[103]
Chickpea	Bromelain hydrolysate	/	11.00 ± 0.80%		790.00 μg/mL	[99]
	Pepsin–pancreatin hydrolysate	/	38.40 ± 1.40%		245.00 μg/mL	
Bambara bean	Alcalase/ thermolysin hydrolysate	/			1.73 mg/mL	[102]
Lupin bean	Hydrolysate (5 mg/mL)	0.4-0.9			100%	[64]
Black bean	Alcalase fraction	/	53.40%	66.10%	96.70%	[95]
Navy bean	Hydrolysate	0.5-3.0	31.00%	> 60.00%	0.10 mg/mL	[101]
Red bean	Hydrolysate	0.5-3.0	36.00%	> 60.00%		[101]
Fermented bean seed	Hydrolysate	3.5-7.0	0.04-0.65 μg/mL			[94]
Common bean	Hydrolysate	/		46.90-50.10%	0.14-0.33 mg/mL	[98]
	Non-hydrolyzed	/			1.20 mg SP/mL	[76]
Germinated Common bean	Hydrolysate	/	7.61-30.88%			[76]
Soybean	Hydrolysate	/		0.05 mg/mL		[62]
Germinated soybean	Pepsin and pancreatin fraction	/	1.70 ± 0.18 mg/mL	2.90 ± 0.07 mg/mL(sucrase)	1.49 ± 0.14 mg/mL	[61]
		> 10.0			1.18 ± 0.15 mg/mL	
		5.0-10.0		2.20 ± 0.40 mg/mL(sucrase)	0.91 ± 0.17 mg/mL	
		< 5.0		1.23 ± 0.19 mg/mL(sucrase)		

SP: Soluble protein
